# Multiple focal atrial tachycardia as a characteristic finding of intractable arrhythmia associated with wild-type transthyretin amyloid cardiomyopathy

**DOI:** 10.1016/j.hrcr.2022.03.010

**Published:** 2022-03-17

**Authors:** Hisanori Kanazawa, Miwa Ito, Yusei Kawahara, Tadashi Hoshiyama, Seiji Takashio, Kenichi Tsujita

**Affiliations:** ∗ Department of Cardiovascular Medicine, Graduate School of Medical Sciences, Kumamoto University, Kumamoto, Japan; † Department of Cardiac Arrhythmias, Kumamoto University, Kumamoto, Japan

**Keywords:** Wild-type transthyretin amyloid cardiomyopathy, Atrial tachycardia, Multiple focal atrial tachycardia, Catheter ablation, Atrioventricular node ablation

**Key Teaching Points**

• Multiple focal atrial tachycardia (AT) is a characteristic finding of wild-type transthyretin amyloid cardiomyopathy (ATTRwt-CM); to our knowledge, this is the first case report on the details of intractable multiple focal AT episodes associated with ATTRwt-CM.
• Especially in elderly patients with ATTRwt-CM, the scar area is widely spread, and multiple focal microreentrant AT episodes are likely to occur in the amyloid-deposited atrium.
• Although multiple focal AT episodes persist, they are readily stopped by catheter contact with the atrial wall during mapping. In contrast, they are not terminated easily by radiofrequency energy application, suggesting that multiple focal AT is fragile but relentless.
• It is difficult to cure multiple focal AT episodes completely because they continue to be induced regardless of the number of radiofrequency energy applications delivered. Therefore, atrioventricular node ablation is a reasonable treatment instead of directly targeting multiple focal AT.

## Introduction

Wild-type transthyretin amyloid cardiomyopathy (ATTRwt-CM) is a secondary cardiomyopathy with cardiac hypertrophy. An increasing number of patients with ATTRwt-CM can be diagnosed by various detailed examinations, such as cardiac magnetic resonance imaging (MRI), technetium-99m-labeled pyrophosphate (^99^mTc-PYP) scintigraphy, and histopathological evidence of amyloid deposition in the tissue, including the abdominal fat or upper gastrointestinal mucosa. In our institution, 129 patients were diagnosed with ATTRwt-CM between December 2002 and December 2019, and we have previously reported the clinical features and prognosis of patients with ATTRwt-CM in the largest number of cases in Japan.^1^

On the other hand, atrial fibrillation (AF), atrial flutter, and atrial tachycardia (AT) are more frequently observed in patients with ATTRwt-CM,^2^, ^3^, ^4^, ^5^ with increasing opportunities for catheter ablation. Under these circumstances, we encountered several cases of ATTRwt-CM with multiple focal AT episodes that were extremely difficult to treat. Although a larger case series of all amyloid subtypes undergoing various atrial arrhythmia ablations has been published,^6^ individual case details of multiple focal AT episodes associated with ATTRwt-CM have not been previously published. Therefore, we report a representative case of multiple focal AT episodes, a characteristic finding of patients with ATTRwt-CM. Written informed consent was obtained from the patient.

## Case report

A 78-year-old man was hospitalized at another hospital for congestive heart failure and was diagnosed with ATTRwt-CM as a result of ^99^mTc-PYP scintigraphy, myocardial biopsy, and genetic testing of transthyretin, which showed no genetic mutation. Hypertrophic cardiomyopathy, ischemic cardiomyopathy, and other secondary cardiomyopathies were excluded by cardiac MRI, coronary angiography, and other examinations and clinical findings. At the time of hospitalization at another hospital, AT had been sustained, but oral administration of 50 mg/day amiodarone, which was prescribed in a very small amount owing to concerns about exacerbation of heart failure, did not restore sinus rhythm even after discharge. The heart failure worsened despite attempted heart rate control therapy. Because AT recurred immediately after cardioversion, the patient was admitted to our hospital for catheter ablation.

**Figure 1:**
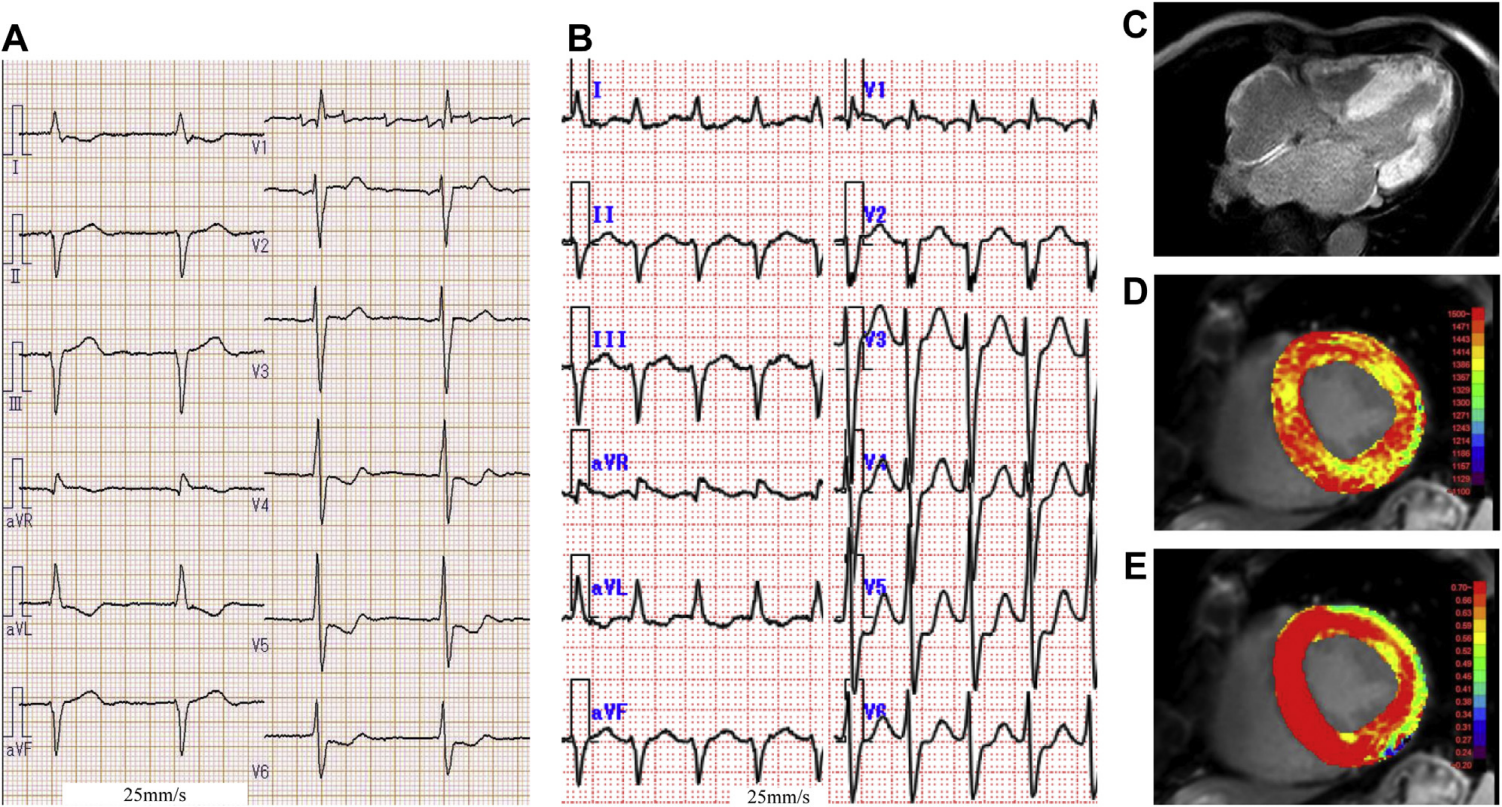
Twelve-lead electrocardiogram (ECG) of atrial tachycardia (AT) and magnetic resonance imaging. **A:** Twelve-lead ECG of AT before admission. **B:** Twelve-lead ECG of AT the time of admission to our hospital. **C–E:** Contrast-enhanced magnetic resonance imaging images. Late gadolinium enhancement was diffusely found in the entire thickened left ventricular wall, right ventricular wall, and left atrial wall (**C**). The native T1 of the thickened interventricular septum was markedly extended to 1444 ms (**D**). Extracellular volume was abnormally high at 89% (**E**).

On a 12-lead electrocardiogram before admission, AT with an atrial rate of 180 beats per minute and an atrioventricular (AV) conduction ratio of 3:1 was sustained (Figure 1A), while another AT with an atrial rate of 155 beats per minute and an AV conduction ratio of 1:1 was sustained at the time of admission to our hospital (Figure 1B). The wall motion of the left ventricle (LV) was reduced owing to the tachycardia, and the ejection fraction decreased to 33%. However, there was concentric hypertrophy of the LV wall on echocardiography (interventricular septal thickness at end-diastole: 15.2 mm; posterior LV wall thickness at end-diastole: 15.8 mm). Furthermore, cardiac contrast MRI also showed concentric hypertrophy of the LV wall, and delayed imaging showed diffuse late gadolinium enhancement in the entire LV and right ventricular wall (Figure 1C). In T1 mapping, native T1 was markedly prolonged to 1444 ms (Figure 1D), and the extracellular volume was abnormally high at 89% (Figure 1E), which were findings of advanced cardiac amyloidosis.

**Figure 2:**
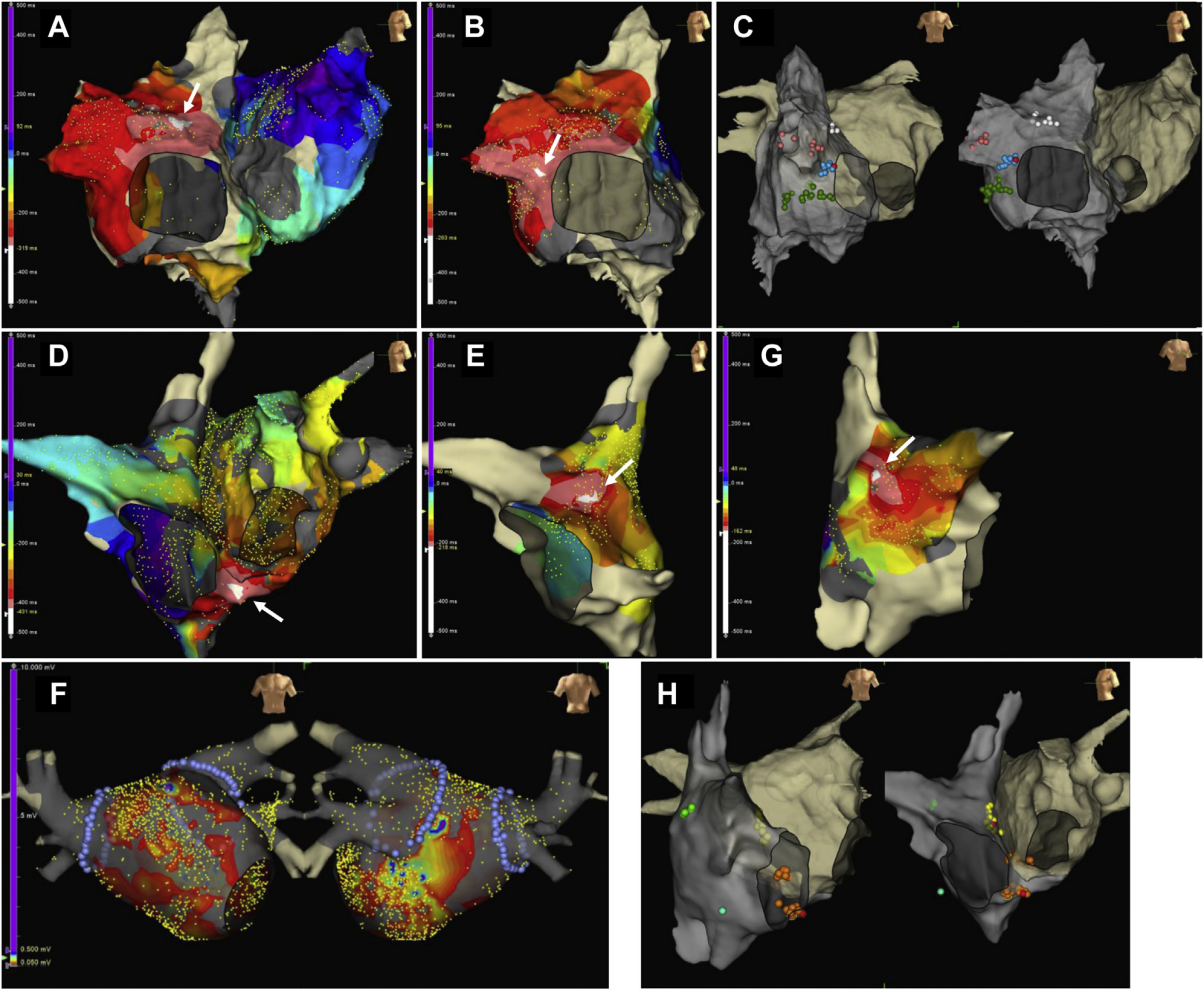
Three-dimensional mapping image of EnSite Precision system (Abbott Laboratories, Abbott Park, IL). **A–C:** Three-dimensional images of the first session at catheter ablation. **D–H:** Three-dimensional images of the second session at catheter ablation. Panels A and B show isochronal mapping of atrial tachycardia (AT) 1 and AT2 (*arrow* indicates the earliest atrial activation site [EAAS]). Panel C shows the ablation point of AT1 (*white*), AT2 (*blue*), AT3 (*dark green*), and AT4 (*rose*). Red rose tag shows the termination point. Panels D, E, and G show isochronal mapping of AT5, AT6, and AT7 (*arrow* indicates the EAAS), respectively. Panel H shows the ablation point of AT5 (*orange*), AT6 (*yellow*), AT7 (*light green*), and AT8 (*aqua*). Red rose tag shows the catheter bump point. Panel F shows the voltage map after bilateral pulmonary vein isolation (*purple*).

Catheter ablation was performed on the second day of hospitalization under conscious sedation using dexmedetomidine in combination with fentanyl. At the start of the session, the same AT (AT1: tachycardia cycle length [TCL] = 418 ms, Figure 1B, Supplemental Figure 1) recorded at the time of admission was sustained. However, during the mapping of AT1 using the Advisor HD Grid catheter of the EnSite Precision System (Abbott Laboratories, Abbott Park, IL), AT1 was easily terminated by the contact of the HD Grid catheter to the wall of the right atrium (RA). AT was then reinduced by burst pacing from the coronary sinus (CS), and another AT2 (TCL = 405 ms, Supplemental Figure 1) was readily induced and persisted. As a result of the mapping, the earliest atrial activation site (EAAS) was found in the RA near the 12 o'clock position of the tricuspid annulus in the left anterior oblique (LAO) view (Figure 2A), and displayed a centrifugal activation pattern that spreads throughout the atria. As AT originating from the AV annulus^7^ was suspected, entrainment pacing was delivered from the high anterolateral RA, high posterolateral RA, high anteroseptal RA, high posteroseptal RA, low anterolateral RA, low posterolateral RA, low posteroseptal RA, and CS ostium. However, although AT was entrained from all 8 sites, the EAAS was not accurately captured orthodromically, suggesting that the mechanism of AT2 was microreentry. Catheter ablation targeting the EAAS was performed using an irrigated contact force–sensing catheter (TactiCath SE, Abbott Laboratories); however, AT2 was not terminated even by the application of radiofrequency energy (RF), including around the EAAS. Then AT2 remapping was conducted; however, AT2 was also unexpectedly terminated by the contact of the HD Grid catheter with the RA wall. Therefore, further reinduction of AT was attempted, and AT1 was induced and sustained.

Because the EAAS of AT1 was also seen in the RA near the 9 o’clock position of the tricuspid valve in the LAO view (Figure 2B) and displayed a centrifugal activation pattern, the pacing of entrainment was performed from the same 7 sites of the RA and CS ostium. However, since the EAAS of AT1 was not orthodromically captured even though entrained from all sites, the RF was applied to the EAAS of AT1, resulting in the extension of the TCL and the termination of AT1. Subsequently, to evaluate the effect of catheter ablation, burst pacing from the CS was performed again, and another AT3 (TCL = 426 ms, Supplemental Figure 1) was immediately induced and sustained. However, AT3 was fragile and terminated during the mapping, and the RF application was delivered to the EAAS of AT3 within the mapping area (the RA near the 8 o’clock position of the tricuspid valve in the LAO view, Figure 2C). In addition, even though another different AT4 (TCL = 455 ms, Supplemental Figure 1) was induced by CS burst pacing, AT4 was also easily terminated by contact with the catheter again. Nevertheless, after RF application to the EAAS of AT4 within the mapping region (RA appendage, Figure 2C), sustained AT was not inducible, and the ablation procedure was concluded.

However, AT with an atrial rate of 110 beats per minute and an AV conduction ratio of 1:1 recurred on the second day after catheter ablation and recurred despite cardioversion. Although the intravenous administration of amiodarone (6-hour loading dose infusion of 48 mg/h followed by maintenance infusion of 25 mg/h) and landiolol were attempted, these were limited by hypotension and cardiogenic shock, requiring intravenous infusion of dobutamine and noradrenaline. For worsening acute renal and liver failure secondary to cardiogenic shock, percutaneous mechanical circulatory support (Impella; Abiomed Inc, Danvers, MA) was initiated. Owing to the restrictive nature of cardiac amyloidosis, both the tachycardia, and the negative inotropic effects of amiodarone and landilol worsened the hemodynamics similarly. Even though cardioversion was performed several times, sinus rhythm could not be maintained and returned to AT or AF rhythm within a few days. Therefore, the second attempt at catheter ablation for AT/AF was conducted on the 85th day of hospitalization under same sedation and analgesics.

As a result of mapping using the HD Grid catheter, the EAAS of tachycardia (TCL = 470 ms, Supplemental Figure 1) was observed in the CS ostium (Figure 2D) and displayed a centrifugal activation pattern. Although differential atrial overdrive pacing was performed,^8^ no ventriculoatrial linking was observed, suggesting that the tachycardia was AT (AT5). Because EAAS of AT5 was entrained but not orthodromically captured, the application of RF was delivered to the EAAS, and focal microreentrant AT5 was finally terminated by the catheter bump to the anterior CS ostium. AT6 (TCL = 721 ms, Supplemental Figure 1) was also easily induced and sustained by the burst pacing from the CS; however, the focal microreentrant AT6 originating from the high anteroseptal RA (Figure 2E) was also terminated by the catheter bump to the EAAS of AT6, and the RF application to the EAAS of AT6 was delivered. Subsequently, a voltage map was created after bilateral pulmonary vein isolation for AF (Figure 2F), but a low-voltage area of 0.05 mV or less was widespread throughout the left atrium. Furthermore, other AT episodes in which the EAAS were likely to exist at a high posterolateral RA (AT7) and low anterolateral RA (AT8) were induced (Figure 2G and 2H and Supplemental Figure 1); however, both AT episodes terminated during the mapping, and RF was applied to the EAAS of these AT episodes. Finally, although not all of the AT episodes could be treated completely and relentless AT episodes were induced by CS burst pacing, tachycardia could be stopped by pacing; therefore, the second ablation procedure was concluded, and the patient was followed up.

**Figure 3:**
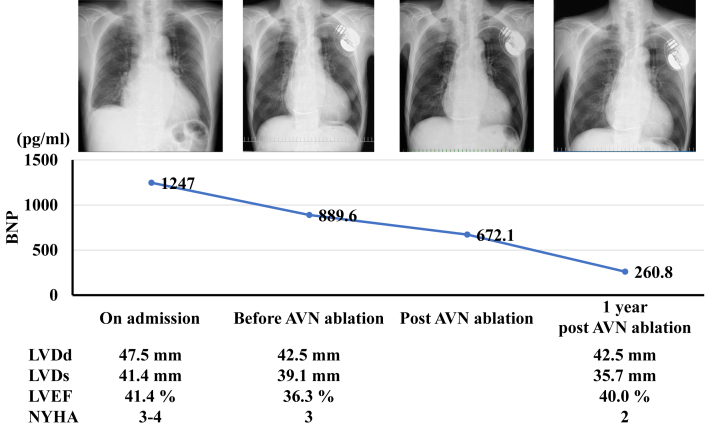
Clinical course after cardiac resynchronization therapy defibrillator and atrioventricular node (AVN) ablation. BNP = brain natriuretic peptide; LVDd = left ventricular end-diastolic dimension; LVDs = left ventricular end-systolic dimension; LVEF = left ventricular ejection fraction; NYHA = New York Heart Association.

However, AT recurred 2 days after the session and recurred after cardioversion; therefore, a cardiac resynchronization therapy (CRT) defibrillator was implanted, and antitachycardia pacing was attempted. Nevertheless, since AT could not be terminated by antitachycardia pacing, AV node ablation was performed on the 129th day of hospitalization. As a result, heart failure could be controlled, and the patient was transferred to the referral hospital. One year later, the patient had not been readmitted to our hospital (Figure 3).

## Discussion

Cardiac amyloidosis is often accompanied by AF and other supraventricular arrhythmias because of amyloid fibril deposits in the extracellular matrix, resulting in an increase in the left atrial pressure owing to diastolic dysfunction associated with LV wall thickening and the conduction dysfunction owing to amyloid deposits in the atrium.^9^ The multiple focal AT that occurred this time was considered reentrant tachycardia because it can be induced and stopped by pacing, and entrained. However, the EAAS was not captured orthodromically regardless of the direction of entrainment pacing, and it seems that there were numerous slow conduction zones everywhere in the atrium.^7^ When the atrial voltage decreases owing to the progression of amyloidosis or aging, multiple focal microreentrant AT episodes may occur,^10^ which we have confirmed in 3 ATTRwt-CM cases, including this case. These patients were 78, 80, and 83 years old, and the tachycardia rate control therapy was also acceptable owing to their age. Therefore, heart failure control can be achieved by performing AV node ablation and CRT defibrillator implantation. Tan and colleagues^6^ reported that AV node ablation is also useful in advanced amyloidosis. It was considered better not to treat too many AT episodes in ATTRwt-CM multiple focal AT cases, because it is difficult to treat all AT episodes even with repeated RF application. Furthermore, in all cases, AT episodes were sustained but easily stopped by the catheter contact to the atrium wall during mapping, whereas AT episodes were not stopped easily by RF application, suggesting that AT episodes are vulnerable but insistent owing to an arrhythmogenic substrate that was widely spread throughout the atrium, which is also characteristic of multiple focal microreentrant AT episodes in ATTRwt-CM. If the presence of ATTRwt-CM is known in advance, it is better not to target for treatment inducible nonclinical AT episodes too much; in that sense, preoperative diagnosis of ATTRwt-CM is very important. Furthermore, Tafamidis treatment was also introduced in this case, but it was discontinued owing to the loss of appetite, loose stools, and weight loss. Tafamidis treatment improves the prognosis of ATTRwt-CM,^11^ meaning that it may be an optional therapy in addition to AV node ablation and CRT in ATTRwt-CM multiple focal microreentrant AT cases in the future.

## Conclusion

In patients with ATTRwt-CM, complete treatment of multiple focal microreentrant AT episodes by ablation is difficult. If multiple focal microreentrant AT episodes are observed in ATTRwt-CM, it is important to consider AV node ablation without excessive treatment for AT.

## Appendix Supplementary data

Supplemental Figure 1 The 12-lead ECG findings in each multiple focal microreentrant atrial tachycardia episodes are shown with the reference of the intracardiac electrocardiogram of the coronary sinus catheter. A difference in the P wave morphology of V1 lead would help to distinguish several atrial tachycardias as unique tachycardias
