# Supplementary material for: Multiple focal atrial tachycardia as a characteristic finding of intractable arrhythmia associated with wild-type transthyretin amyloid cardiomyopathy
Source: HeartRhythm Case Rep. 2022 Mar 17;8(6):420–4. doi: 10.1016/j.hrcr.2022.03.010 (PMC9237377; doi:10.1016/j.hrcr.2022.03.010)
Supplement: Supplemental Figure 1 — The 12-lead ECG findings in each multiple focal microreentrant atrial tachycardia episodes are shown with the reference of the intracardiac electrocardiogram of the coronary sinus catheter. A difference in the P wave morphology of V1 lead would help to distinguish several atrial tachycardias as unique tachycardias [file mmc1.pptx]

## Slide 1
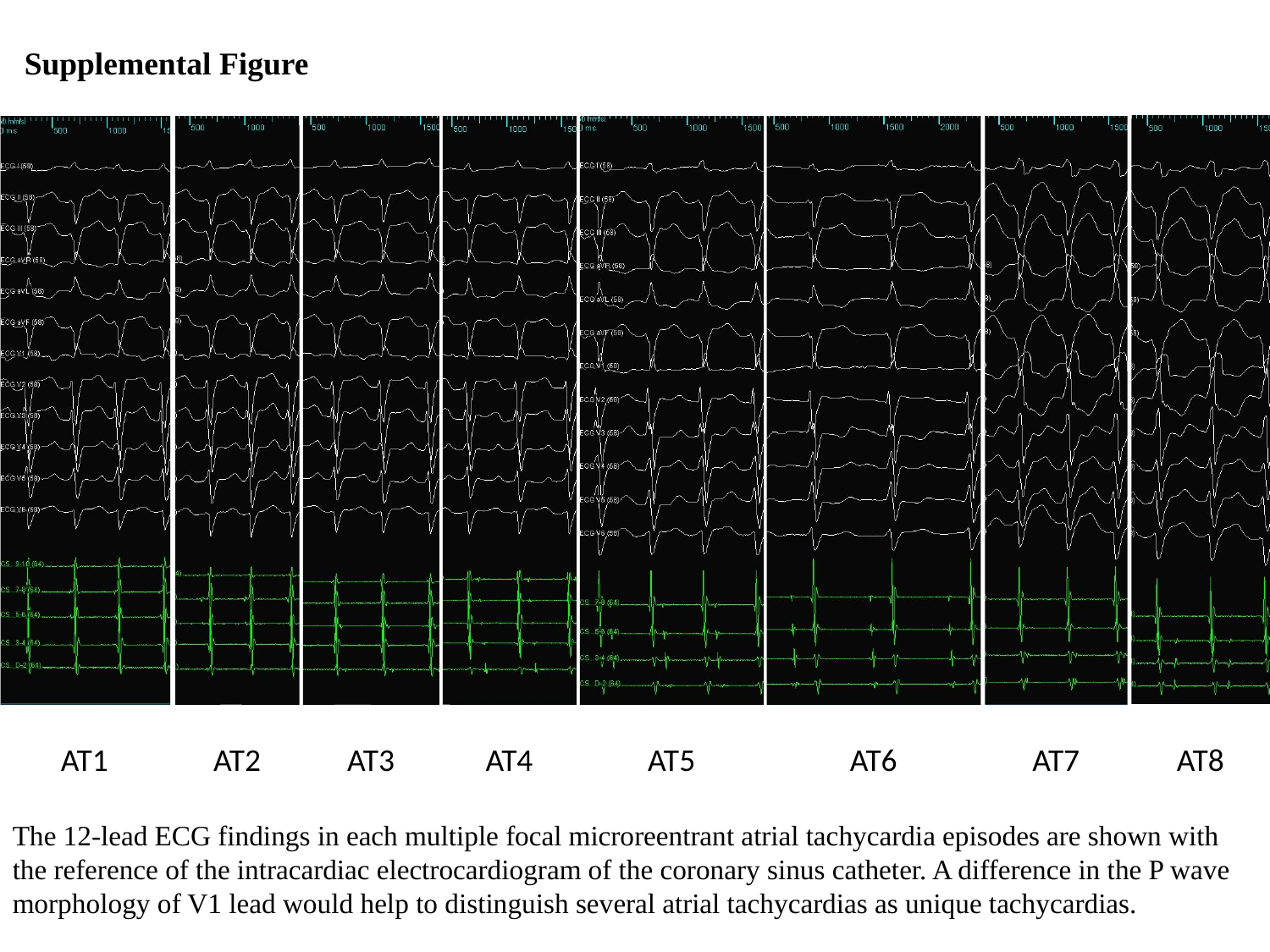

Supplemental Figure
AT5
AT6
AT7
AT8
AT1
AT2
AT3
AT4
The 12-lead ECG findings in each multiple focal microreentrant atrial tachycardia episodes are shown with the reference of the intracardiac electrocardiogram of the coronary sinus catheter. A difference in the P wave morphology of V1 lead would help to distinguish several atrial tachycardias as unique tachycardias.
